# Semi-analytical hierarchical Bayesian inference of nonlinear model structure in stochastic dynamics: Applied to compartmental models of infectious diseases

**DOI:** 10.1371/journal.pone.0350747

**Published:** 2026-07-10

**Authors:** Brandon Robinson, Philippe Bisaillon, Rimple Sandhu, Mohammad Khalil, Jodi D. Edwards, Tetyana Kendzerska, Thomas Walker, Shirley Mills, Chris Pettit, Dominique Poirel, Abhijit Sarkar

**Affiliations:** 1 Department of Civil and Environmental Engineering, Carleton University, Ottawa, Ontario, Canada; 2 University of Ottawa Heart Institute, Ottawa, Ontario, Canada; 3 Computational Science Center, National Laboratory of the Rockies, Golden, Colorado, United States of America; 4 Quantitative Modeling & Analysis Department, Sandia National Laboratories, Livermore, California, United States of America; 5 School of Epidemiology and Public Health, University of Ottawa, Ottawa, Ontario, Canada; 6 ICES, Ottawa, Ontario, Canada; 7 The Ottawa Hospital Research Institute, Ottawa, Ontario, Canada; 8 Department of Medicine, Faculty of Medicine, Division of Respirology, University of Ottawa, Ottawa, Ontario, Canada; 9 School of Mathematics and Statistics, Carleton University, Ottawa, Ontario, Canada; 10 Aerospace Engineering Department, United States Naval Academy, Annapolis, Maryland, United States of America; 11 Department of Mechanical and Aerospace Engineering, Royal Military College of Canada, Kingston, Ontario, Canada; Northwest Normal University, CHINA

## Abstract

A Bayesian computational framework for parsimonious inference in stochastic nonlinear dynamical systems is presented. This framework enables the concurrent estimation of system states, time-varying parameters, time-invariant parameters, and the optimal sparsity structure of the model parameters. Because differential equation-based models are often simplified mechanistic or phenomenological representations, robust inference from noisy measurement data requires explicit treatment of model error and uncertainty. Model error and time-varying parameters can be represented as random processes, enabling inference while making minimal assumptions about the underlying sources of discrepancy and variability. Adopting stochastic differential equation representations affords the model significant flexibility, but can also render it susceptible to overfitting during statistical inversion, where the inferred model may track noise rather than the underlying signal. To alleviate the effects of overfitting and to enable the discovery of the optimal sparse representation of the time-invariant parameters, a Bayesian sparse learning algorithm is embedded within the framework. This sparse learning framework adopts an approximate hierarchical Bayesian setting defined by a series of semi-analytical expressions. The model structure inference framework is validated using a stochastic compartmental model for tracking and forecasting active cases of an infectious disease. Compartmental models describe population-level infectious disease dynamics through interactions among population fractions grouped by disease state. Mathematically, such models consist of a system of coupled ordinary differential equations. This example adopts an expressive compartmental model that includes multiple possible interactions between disease states, motivated by early uncertainty surrounding COVID-19 reinfection dynamics and their implications for long-term epidemic forecasting. The sparse learning exercise permits the inference of *a priori* unknown epidemiological dynamics from simulated public health data, discovering the nested compartmental model that optimizes the trade-off between average data-fit and model complexity. It is shown that inducing sparsity among the model parameters eliminates redundant interactions between compartments, equivalently revealing the optimal coupling structure between differential equations.

## Introduction

Mechanistic models are widely used to describe the dynamics of complex systems in science and engineering. Such models are inevitably simplified representations of the physical phenomena they describe. For these physics-based models, handling uncertainty in the appropriate level of model complexity explicitly leads to a model selection problem. Whereas data-driven sparse learning applied to purely empirical models searches libraries of candidate functions, when applied to a physically-grounded mechanistic model, it permits the inference of the optimal nested model, such that any sparse combination of terms retains a physically meaningful interpretation. Model discovery has been explored for efficient data-optimal model selection in a number of contexts, popularized by its application to dynamical systems, namely the sparse identification of nonlinear dynamics (SINDy) algorithm [1–3]. This algorithm can be viewed as a reformulation of the least absolute shrinkage and selection operator (LASSO) in which the standard regularized regression problem is reframed from a static algebraic mapping between inputs and outputs to sparse regression of state derivatives onto candidate nonlinear functions of the state variables. This setup permits the estimation of coefficients associated with dynamically relevant terms while driving irrelevant coefficients towards zero, thereby sparsifying the parameter space. The learned sparsity structure is then directly interpreted as the discovered system of governing equations. The SINDy algorithm has been used in many applications, including compartmental models [4,5], where sparse learning is used to rediscover the differential equation structure and associated terms from a large library of candidate functions consisting of candidate nonlinear combinations of the model states. However, this approach is notably limited by the requirement that the full state space be observed, which is generally unrealistic, or by the need to otherwise impose constraints to enforce the ostensibly unknown dynamics. Furthermore, this approach fails to accommodate the possibility of time-varying parameters, except in the case of a rigid parametric representation thereof. Another general limitation of the SINDy algorithm and similar regularized regression approaches to model discovery is their reliance on frequentist statistics, which provides only point estimates and does not quantify uncertainty. Extensions have been developed to enable a Bayesian implementation [6] and to handle stochastic systems [7]. These limitations motivate a Bayesian sparse learning approach that operates directly on the likelihood function, which can be constructed from only a partial observation of the model states. By embedding sparsity within a robust estimation framework, this method can concurrently infer relevant model parameters, observable and latent model states and time-varying parameters under stochastic excitation.

The nonlinear sparse Bayesian learning (NSBL) algorithm was introduced by Sandhu et al. [8,9] as a conceptual extension to the sparse Bayesian learning (SBL) [10]. This extension critically relaxed several limiting assumptions of the SBL algorithm, permitting the use of non-Gaussian likelihood functions and parameter prior probability density functions (pdfs). Thus, the NSBL algorithm is theoretically applicable to any nonlinear mapping for which the likelihood function can be computed. Due to its generality, the algorithm has wide-ranging applications, including dynamical systems [11] and black-box models such as Bayesian neural networks [12]. In these applications, it has been demonstrated that the NSBL algorithm functions as a computationally efficient mechanism for Bayesian model selection. Dabiran et al. [13] offer additional insights into the hierarchical structure of the NSBL algorithm, showing it to be both an efficient model selection method and a tractable approximation of full hierarchical Bayesian inference.

The generality of the NSBL algorithm is leveraged by embedding it within a Bayesian computational framework [14–16] for combined state and parameter estimation in stochastic nonlinear dynamical systems. This framework provides the likelihood function required by NSBL. It additionally permits the demarcation between parameters that are known (or thought) to vary in time and parameters that are static in the model, broadening the class of problems to which the framework can be applied. This distinction affects how parameters are treated in the joint inference procedure. While time-invariant parameters are jointly estimated as static quantities, the time-varying parameters are estimated recursively alongside the system states, which themselves vary in time according to the system dynamics [15,16]. In contrast to standard approaches to joint state and parameter estimation, this distinction between time-varying and time-invariant parameters helps avoid introducing artificial dynamics into the system which would otherwise occur if all parameters were appended to the system states for the purpose of inference. Separating the estimation of the time-invariant parameters reduces the dimensionality of the augmented state, limiting the degree of nonlinearity introduced by parameter-state interactions in the augmented system. Furthermore, this arrangement permits the robust estimation of important model features (e.g., the process noise strength and initial conditions) as time-invariant parameters [15,16]. However, the flexibility afforded to the model by permitting parameters to vary in time makes it susceptible to overfitting the data. This creates an ideal scenario for applying the NSBL algorithm.

In the *Materials and methods* section, the Bayesian formulation of the inference problem is expressed to accommodate the semi-analytical hierarchical Bayesian structure of the NSBL algorithm. This includes the derivation of a conditional parameter posterior pdf and a general expression for the likelihood function for stochastic dynamical systems. This is followed by a brief overview of the NSBL algorithm, which ultimately enables the model structure inference, operating on the aforementioned likelihood function. This arrangement leads to the generality of this framework for nonlinear stochastic dynamical systems, avoiding the need for *ad hoc* application dependent formulations. In the *Results* section, the performance of the framework is demonstrated for a stochastic compartmental model from the field of infectious disease modelling. Here, the data-optimal interconnectivity of the compartments is discovered, using synthetic infection data generated from a randomly fluctuating infection rate parameter. In addition to parametric uncertainties, structural uncertainty relating to model selection has been shown to have a significant effect in epidemiological studies [17]. The COVID-19 pandemic highlighted the critical role of model selection under uncertainty in public health forecasting, where modelling decisions hinging on questions such as whether recovered individuals could be reinfected and at what time-scale, could have profound implications for predicted case trajectories. Hence, the inclusion of sparse learning within the estimation framework permits the automatic model structure discovery by pruning redundant parameters, thereby decoupling interconnected models by eliminating coupling terms in a system of differential equations. This naturally guides the state and parameter inference towards the values associated with the data-optimal nested compartmental model. The experiment uses synthetic data to illustrate the ability of the framework to capture the data-generating model among a number of possible sparse representations of the dynamics [18,19].

The principal objective of this work is to formulate and demonstrate the proposed approach for model structure inference for stochastic dynamical systems. A Bayesian sparse learning algorithm for general nonlinear mappings is embedded within a framework for joint state and parameter estimation, as outlined in the mathematical preliminaries. The proposed model structure inference approach is evaluated in an infectious disease modelling application using synthetic data. In this setting, with a known ground truth, the framework successfully infers the observable and latent states and time-varying parameters, and the values and sparsity structure of the time-invariant parameters, thereby achieving the stated objective.

## Materials and methods

Model structure inference is achieved through sparse learning, addressing the joint objectives of quantifying uncertainty and alleviating overfitting in mechanistic models. The model structure inference is driven by nonlinear sparse Bayesian learning (NSBL), a Bayesian sparse learning algorithm that plays a conceptually analogous role to regularized regression methods used in frequentist approaches of model discovery. NSBL operates in a semi-analytical hierarchical Bayesian setting, in which data-dependent hierarchical priors provide a mechanism for inducing sparsity in the parameter space.

The concurrent estimation of time-varying parameters, time-invariant parameters, and the data-optimal nested model structure is achieved using type-II maximum *a posteriori* (MAP) estimation [20]. While type-I MAP estimation identifies the parameter values that maximize the parameter posterior, type-II MAP instead identifies the values of the hyperparameters, namely the parameters of the hierarchical priors, that maximize the hyperparameter posterior. This procedure can be equivalently interpreted as maximizing the regularized model evidence, optimally balancing the trade-off between average data-fit and model complexity. By fixing the hyperparameters to their optimized values, type-II MAP retains and quantifies uncertainty in the parameters through their conditional posterior pdfs, enabling joint estimation of states and parameters associated with the data-optimal model structure.

The NSBL algorithm critically operates on the likelihood function, which underpins its broad applicability. In the subsections that follow, the estimation framework used to compute the likelihood function for a stochastic dynamical system containing both time-varying and time-invariant parameters is presented. Subsequently, for completeness, a high-level description of the NSBL algorithm is provided, elucidating the efficient and scalable type-II MAP estimation that is enabled by a series of semi-analytical expressions.

### Estimation framework

First, the notation for the general discrete state space representation of nonlinear stochastic dynamical systems is defined. The time-evolution of the system states are governed by the model operator, which is a discrete representation of the continuous system. In order to append the time-varying parameters to the original system states, an additional differential equation model is defined for the time-varying parameters, whereby their dynamics are inflated by an artificial random noise process. This allows for recursive estimation through a state estimation procedure which can be performed using a variety of nonlinear filters, such as the Extended Kalman Filter [21–23], the Ensemble Kalman Filter [24–26], or the Particle Filter [27–30]. The estimation of time-invariant parameters may be achieved using sampling-based methods like Markov chain Monte Carlo (MCMC) [31,32], optimization-based approximations like variational inference [33,34] or multi-modal Laplace approximation-based representations [35,18]. Though the estimation of the time-invariant parameters and the systems states are performed using two different methods, it is important to note that the computational framework provides the joint posterior of the system states, time-varying parameters and time-invariant parameters.

For time-invariant parameters, $\phi_{s}$, original system states, $\{{𝐮}_{k}{\}}_{k=0}^{K}$, and time-varying parameters, $\{\phi_{t(k)}{\}}_{k=0}^{K}$, indexed by *k* at discrete instances of time, the state space representation is expressed as [14–16,36],


$$\begin{array}{c} {𝐮}_{k+1}=g_{k}({𝐮}_{k},\phi_{t(k)},\phi_{s},{𝐪}_{k}^{u}),\;k=1,\ldots ,K \end{array}$$ (1)

$$\begin{array}{c} \phi_{t(k+1)}=f_{k}(\phi_{t(k)},{𝐪}_{k}^{\phi }), \end{array}$$ (2)

where model operators *g*_*k*_ and *f*_*k*_ define the the system dynamics and the dynamics of the time-varying parameters, respectively. Process noise terms ${𝐪}_{k}^{u}$ and ${𝐪}_{k}^{\phi }$ represent model error in the system dynamics and an artificial noise process that perturbs the dynamics of the time-varying parameters, respectively. Augmenting the state vector as ${𝐱}_{k}=\{{𝐮}_{k},\phi_{t(k)}{\}}^{T}$ and the model error as ${𝐪}_{k}=\{{𝐪}_{k}^{u},{𝐪}_{k}^{\phi }{\}}^{T}$, the augmented stochastic nonlinear system can now be described succinctly as [14–16,36],


$$\begin{array}{c} {𝐱}_{k+1}={𝐠}_{k}({𝐱}_{k},\phi_{s},{𝐪}_{k}), \end{array}$$ (3)

where the augmented model operator is denoted **g**_*k*_ and the model error is **q**_*k*_. The set of time-invariant parameters, $\phi_{s}$, consist of static parameters of the original dynamical system and may additionally contain the parameters of the augmented system, the model error such as the process noise strength, and initial conditions such as the mean vector of the initial state pdf.

The measurement operator relates the data to direct or indirect observations of a subset of the system states at discrete time instances coinciding with the computation grid as defined by [14–16,36],


$$\begin{array}{c} {𝐝}_{j}={𝐡}_{j}({𝐱}_{d(j)},\phi_{s},\epsilon_{j}),\;j=1,\ldots ,J \end{array}$$ (4)

where incoming observations are indexed by *k* = *d*(*j*) indicating that the *j*th data point coincides with the *k*th time step, and the measurement noise is represented by $\epsilon_{j}$.

The ultimate inferential objective is to compute the joint posterior pdf of the augmented state and time-invariant parameters conditioned on the observations $𝒟=\{{𝐝}_{j}{\}}_{j=1}^{J}$ and hyperparameters, α, defined as,


$$\begin{array}{c} \text{p}({𝐱}_{0},\ldots ,{𝐱}_{K},\phi_{s}|𝒟,\alpha )=\text{p}({𝐱}_{0},...,{𝐱}_{K}|\phi_{s},𝒟)\text{p}(\phi_{s}|𝒟,\alpha ), \end{array}$$ (5)

where $\text{p}({𝐱}_{0},...,{𝐱}_{K}|\phi_{s},𝒟)$ is the conditional posterior pdf of the augmented state and $\text{p}(\phi_{s}|𝒟,\alpha )$ is the conditional posterior pdf of the time-invariant parameters. Unrolling the conditional state pdfs, Eq. (5) can be expanded as [14–16,36],


$$\begin{array}{cc} \text{p}({𝐱}_{0},\ldots ,{𝐱}_{d(1)},\ldots ,{𝐱}_{d(J)}, & \ldots ,{𝐱}_{K},\phi_{s}|𝒟,\alpha )\\ \propto \text{p}(\phi_{s}|\alpha )\text{p}({𝐱}_{0}|\phi_{s}) & \text{p}({𝐱}_{1}|{𝐱}_{0},\phi_{s})\text{p}({𝐱}_{2}|{𝐱}_{1},\phi_{s})\ldots \text{p}({𝐱}_{d(1)}|{𝐱}_{d(1)-1},\phi_{s})\text{p}({𝐝}_{1}|{𝐱}_{d(1)},\phi_{s})\\ & \;\vdots \\ & \text{p}({𝐱}_{d(J-1)+1}|{𝐱}_{d(J-1)},\phi_{s})\ldots \text{p}({𝐱}_{d(J)}|{𝐱}_{d(J)-1},\phi_{s})\text{p}({𝐝}_{J}|{𝐱}_{d(J)},\phi_{s})\\ & \text{p}({𝐱}_{d(J)+1}|{𝐱}_{d(J)},\phi_{s})\ldots \text{p}({𝐱}_{K}|{𝐱}_{K-1},\phi_{s}), \end{array}$$ (6)

where $\text{p}(\phi_{s}|\alpha )$ is the conditional prior pdf of the time-invariant parameters, $\text{p}({𝐱}_{0}|\phi_{s})$ is initial state pdf, expressed conditionally to accommodate the parametric estimation of the initial conditions. $\text{p}({𝐱}_{k}|{𝐱}_{k-1},\phi_{s})$ is the conditional augmented state pdf, and $\text{p}({𝐝}_{j}|{𝐱}_{d(j)},\phi_{s})$ is the likelihood of the *j*th observation. Upon marginalizing Eq. (6) over the augmented state, the following expression for the unnormalized conditional posterior pdf of the time-invariant parameters remains,


$$\begin{array}{cc} \text{p}(\phi_{s}|𝒟,\alpha ) & \propto \underset{\text{prior}}{\underbrace{.\text{p}(\phi_{s}|\alpha )}}\underset{\text{likelihood function}}{\underbrace{\prod_{j=1}^{J}[\int_{-\infty }^{\infty }\text{p}({𝐱}_{d(j)}|{𝐱}_{d(j)-1},\phi_{s})\text{p}({𝐝}_{j}|{𝐱}_{d(j)},\phi_{s})\text{d}{𝐱}_{d(j)}]}},\\ & \propto \text{p}(\phi_{s}|\alpha )\text{p}(𝒟|\phi_{s}), \end{array}$$ (7)

where the likelihood function, $\text{p}(𝒟|\phi_{s})$, is the product of the likelihood computed at each data point, **d**_*j*_ under the assumption that the measurements are conditionally independent given the augmented state ${𝐱}_{d(j)}$. The computation of the likelihood can therefore be performed using a variety of nonlinear filters [14–16,36]. The current implementation leverages the extended Kalman filter (EKF) to compute the likelihood function in Eq. (7) and the conditional posterior of the augmented state on the right-hand-side of Eq. (5).

Note that the likelihood function is independent of the hyperparameters, α. This property enables efficient type-II MAP estimation within NSBL, as the computationally expensive likelihood function computation is only performed once and subsequently reused throughout the iterative, comparatively inexpensive hyperparmeter optimization procedure.

### Nonlinear sparse Bayesian learning

The sparsity-inducing priors used in the NSBL algorithm are automatic relevance determination (ARD) priors [37]. Each ARD prior is an independent zero-mean Gaussian distributions with a variable precision parameter, the aforementioned hyperparameters α. The hyperparameters themselves have priors, referred to as hyperpriors, representing an increase in the level of hierarchy with respect to the standard Bayesian inference setup. The optimal sparsity structure is inferred through the optimal selection of the hyperparameters, which govern the relevance or irrelevance of its associated model parameter [9,10].

The NSBL setup begins with the expression for the posterior pdf of the unknown model parameters ϕ conditioned on the data 𝒟 and hyperparameters α [9],


$$\begin{array}{c} \text{p}(\phi_{s}|𝒟,\alpha )=\frac{\text{p}(\phi_{s}|\alpha )\text{p}(𝒟|\phi_{s})}{\text{p}(𝒟|\alpha )}\propto \text{p}(\phi_{s}|\alpha )\text{p}(𝒟|\phi_{s}), \end{array}$$ (8)

where p(𝒟|α) is the model evidence. At this point, the model evidence can simply be considered as a normalizing constant, which permits the rediscovery of Eq. (7). However, the model evidence plays a significant role in the optimal selection of the hyperparameters as outlined later.

The NSBL algorithm operates on three main principles, namely

1. the introduction of a **hybrid prior** where parameters with known relevance are assigned informative priors and the remainder are assigned automatic relevance determination (ARD) priors,
2. the construction of a **mixture model approximation** of the unnormalized density given by the product of the likelihood function and the informative priors,
3. the **optimization of the hyperparameters**, which leverages the semi-analytical expressions available through NSBL.

#### Hybrid prior.

The NSBL algorithm operates by first deconstructing the vector of time-invariant parameters as $\phi_{s}=\{\phi_{\alpha },\phi_{-\alpha }\}$, distinguishing between parameters that are *a priori* relevant, $\phi_{-\alpha }$, and parameters whose relevance is questionable, $\phi_{\alpha }$. The *a priori* relevant parameters consist of parameters for which either there is some prior mechanistic knowledge or that the modeller simply does not wish to have removed from the model by NSBL. These parameters are assigned known priors, $\text{p}(\phi_{-\alpha })$. The questionable parameters are the set of time-invariant parameters among which the modeller would like to induce sparsity. Sparsity-inducing priors, $\text{p}(\phi_{\alpha }|\alpha )$, are used to this end. As in SBL [10], Gaussian ARD priors are utilized, such that,


$$\begin{array}{c} \text{p}(\phi_{\alpha }|\alpha )=𝒩(\phi_{\alpha }|\text{0},{\text{A}}^{-1}). \end{array}$$ (9)

This prior is a zero-mean normal distribution with a variable precision matrix, A=diag(α). The diagonal entries are unique variable precision hyperparameters $\alpha_{i}$ corresponding to each questionable parameter $\phi_{i}\in \phi_{\alpha }$ such that $\text{p}(\phi_{i}|\alpha_{i})=𝒩(\phi_{i}|0,\alpha_{i}^{-1})$. Low precision, ($\alpha_{i}\to 0$) makes the prior akin to a non-informative Gaussian prior on the associated parameter, $\phi_{i}$. Conversely, high precision ($\alpha_{i}\to \infty$) makes the prior behave like a Dirac delta function, forcing the parameter towards zero, due to the fixed mean of the ARD prior.

If the *a priori* relevant parameters are given arbitrary priors $\text{p}(\phi_{\text{-}\alpha })$ which are independent of hyperparameters, α, the joint prior pdf is


$$\begin{array}{c} \text{p}(\phi |\alpha )=\text{p}(\phi_{\text{-}\alpha })\text{p}(\phi_{\alpha }|\alpha )=\text{p}(\phi_{\text{-}\alpha })𝒩(\phi_{\alpha }|\text{0},{\text{A}}^{-1}). \end{array}$$ (10)

Thus, substituting the above expression for the hybrid prior, the expression for the unnormalized posterior becomes,


$$\begin{array}{c} \text{p}(\phi_{s}|𝒟,\alpha )\propto \text{p}(𝒟|\phi_{s})\text{p}(\phi_{\text{-}\alpha })𝒩(\phi_{\alpha }|\text{0},{\text{A}}^{-1}). \end{array}$$ (11)

#### Mixture model approximation.

Noting that both the known prior and the likelihood function in Eq. (11) are both independent of α, the key to the efficiency of the algorithm is achieved by approximating the product of the likelihood function and known prior by a Gaussian mixture model (GMM), such that the numerator of Eq. (8) is given by a sum of products of Gaussian distributions.


$$\begin{array}{c} \text{p}(𝒟|\phi_{s})\text{p}(\phi_{-\alpha })\approx \sum_{k=1}^{K}a^{\left(k\right)}𝒩(\phi_{s}|\mu^{\left(k\right)},\Sigma^{\left(k\right)}), \end{array}$$ (12)

where, *a*^(*k*)^, $\mu^{\left(k\right)}$, and $\Sigma^{\left(k\right)}$ are the kernel weight, mean vector, and covariance matrix of kernel *k*, respectively. Adopting a GMM-based representation of the product of the likelihood function and the known prior, rather than a Gaussian approximation thereof, for instance, allows for non-Gaussian effects to be captured while retaining the conjugacy between the Gaussian ARD prior and the individual kernels of the GMM. This conjugacy is exploited to derive several semi-analytical expressions for Bayesian entities in terms of the kernel parameters of the GMM and the hyperparameters. These include the parameter posterior, $\hat{\text{p}}(\phi_{s}|𝒟,\alpha )$, model evidence, $\hat{\text{p}}(𝒟|\alpha )$, the sparse learning objective function and its associated gradient vector and Hessian matrix (see [9,11]).

Following [8,9,11], the kernel parameters are obtained by first sampling from the unnormalized density $\text{p}(𝒟|\phi_{s})\text{p}(\phi_{-\alpha })$, where the likelihood function is computed according to Eq. (7). This implementation first uses MCMC with nested EKF routine to generate samples from the posterior of the unnormalized density in Eq. (12). Subsequently uses kernel density estimation (KDE) to preserve the non-Gaussian features captured by the sampling algorithm while computing the kernel parameters. Note, however, that the framework is amenable to lower-dimensional GMM approximations, and sampling-free methods, but critically rely on nested state estimation to evaluate the likelihood function because of the inherent uncertainty in the state pdf [18].

#### Optimization of the hyperparameters.

Recall, the GMM in Eq. (12) is independent of the hyperparameters. Hence, the kernel parameters are fixed during the optimization of α. The optimal values of $\alpha_{i}$ are obtained by maximizing the hyperparameter posterior,


$$\begin{array}{c} \text{p}(\alpha |𝒟)=\frac{\text{p}(𝒟|\alpha )\text{p}(\alpha )}{\text{p}(𝒟)}\propto \text{p}(𝒟|\alpha )\text{p}(\alpha ), \end{array}$$ (13)

where the denominator, p(𝒟), is a normalization constant that is analytically intractable in general, and p(α) is the hyperprior,


$$\begin{array}{c} \text{p}(\alpha )=\prod_{i=1}^{N_{\alpha }}\text{Gamma}(\alpha_{i}|r_{i},s_{i}), \end{array}$$ (14)

where $N_{\alpha }$ denotes the number of questionable parameters, and *r*_*i*_ and *s*_*i*_ are the rate and shape parameters of the Gamma distribution, respectively. A Gamma hyperprior with fixed rate and shape parameters is used due to its conjugacy with the Gaussian kernels of the mixture model representation of the model evidence. The conditional Gaussian prior with a Gamma hyperprior becomes a Student’s t-distribution upon marginalizing over the hyperparameters. Gaussian distributions are not considered to be sparsity-inducing priors when their precision or variance are fixed (a Bayesian analog to *L*_2_ regularization), unlike the Laplace prior (a Bayesian analog to *L*_1_ regularization) [20]. However, with the marginal parameter pdf being a Student’s t-distribution, the heavy tails and a high concentration of probability density near zero makes it suitable for encouraging sparsity among the parameters.

As mentioned previously, in contrast to full hierarchical Bayesian inference, a point estimate of the hyperparameters is obtained rather than the full posterior density thereof. A type-II ML optimizes the model evidence, whereas type-II MAP optimizes the hyperparameter posterior, thereby including the normalizing properties of the hyperprior [20]. Considering a type-II MAP estimate amounts to maximizing of the product of two terms in the numerator, the model evidence p(𝒟|α) and the hyperprior p(α), and discarding the often intractable normalizing constant p(𝒟) in the denominator,


$$\begin{array}{c} \alpha^{\text{map}}=\underset{\alpha }{\mathrm{arg max}}\{\text{p}(\alpha |𝒟)\}=\underset{\alpha }{\mathrm{arg max}}\{\text{p}(𝒟|\alpha )\text{p}(\alpha )\}. \end{array}$$ (15)

The optimization is performed in log-space for both computation and analytical convenience. The sparse learning objective therefore becomes the value of logα which maximizes the sum of the log evidence and log-hyperprior. The objective function therefore becomes [11],


$$\begin{array}{c} \log\alpha^{\text{map}}=\underset{\log\alpha }{\mathrm{arg max}}(\log\hat{\text{p}}(𝒟|\log\alpha )+\sum_{i=1}^{N_{\alpha }}(r_{i}\log\alpha_{i}-s_{i}\alpha_{i})), \end{array}$$ (16)

where $\hat{\text{p}}(𝒟|\log\alpha )$ is the GMM-based estimate of the model evidence and the summand are the terms that remain from the Gamma hyperprior after taking the natural logarithm and discarding terms independent of α [8,9].

#### Conditional posterior.

The NSBL methodology permits the efficient computation of a GMM-based approximation of the conditional posterior p(ϕ|𝒟,α) in Eq. (7) as derived in [8,9],


$$\begin{array}{c} \text{p}(\phi_{s}|𝒟,\alpha )\approx \sum_{k=1}^{K}w^{\left(k\right)}𝒩(\phi_{s}|{𝐦}^{\left(k\right)},{𝐏}^{\left(k\right)}), \end{array}$$ (17)

where the mixture weights, *w*^(*k*)^, mean vectors **m**^(*k*)^, and **P**^(*k*)^ are available analytically as a function of the fixed mixture parameters *a*^(*k*)^, $\mu^{\left(k\right)}$ and $\Sigma^{\left(k\right)}$ from the GMM in Eq. (12) and the variable hyperparameters, α. For the exact expressions, see Eqs. (10)–(12) and (B.6–B.14) in Ref. [9]. The parameter posterior pdf of the optimal sparse model is thus obtained by conditioning the expression in Eq. (17) on the optimal hyperparameters, $\alpha^{\text{map}}$.

Substituting this GMM-based approximation of the conditional posterior in Eq. (5) permits the computation of the full joint posterior of the states, time-varying parameters, and time-invariant parameters. The posterior of the augmented state vector can be obtained by marginalizing over the time-invariant parameters. For simplicity, the marginal state pdfs shown in the results that follow are conditioned on the MAP estimate of the conditional posterior of the time-invariant parameters.

## Results and discussion

To evaluate the practical implementation of the model structure inference framework in stochastic dynamical systems, consider an example from infectious disease modelling using compartmental models and synthetic data. Practical limitations surrounding data collection and reporting during epidemics and pandemics naturally lead to measurements that are noisy and incomplete observations of the model states. Furthermore, introducing stochasticity in the model accounts for the time-varying and randomness inherent to population-level disease transmission.

This numerical investigation is designed to mimic early pandemic modelling where data sources supporting these efforts provided limited information to researchers, such as aggregated daily case counts. Reminiscent of early COVID-19 modelling, this setup considers uncertainty surrounding the impact of possible reinfection on long-term pandemic trends [38]. This experiment reveals the impact of misspecified models, before demonstrating the proposed framework’s ability to correctly infer the states, parameters, and model structure of the data-generating model, enabled by sparse learning.

### Model operator

**Fig 1:**
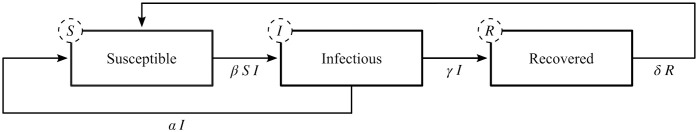
A fully-connected SIRS model. Flowchart depicting the fully connected SIRS model in Eq. (18).

Consider an extension of the fundamental SIR epidemic model (S→I→R), comprising susceptible *S*, infectious *I*, and recovered *R* compartments [39,40]. As depicted in Fig 1, this extension introduces two additional reinfection mechanisms beyond the baseline SIR model, with and without a period of temporary immunity (I→R→S→I and I→S→I, respectively). This model is described mathematically by the system of nonlinear coupled ordinary differential equations in Eq. (18). This model will be referred to as a *fully-connected SIRS model* in order to distinguish it from the more typical representation of the SIRS model that follows in Eq. (21),


$$\begin{array}{c} \frac{dS}{dt}=-\beta SI+aI+\delta R, \end{array}$$ (18a)

$$\begin{array}{c} \frac{dI}{dt}=\beta SI-aI-\gamma I, \end{array}$$ (18b)

$$\begin{array}{c} \frac{dR}{dt}=\gamma I-\delta R. \end{array}$$ (18c)

The three compartments are interconnected in such a way that, nested within the model in Eq. (18), one can obtain standard representations of the SIR model in Eq. (19), the SIS model in Eq. (20) and the SIRS model in Eq. (21) [40]. The interconnectivity of the compartments in Eq. (18) implies that some prior knowledge or intuition on the disease dynamics is imposed. In this way, the model discovery is not entirely naive *a priori*; it permits the automatic discovery of the data-optimal nested model while enforcing critical constraints, such as the conservation of the population, and logically consistent disease progressions.

**Fig 2:**
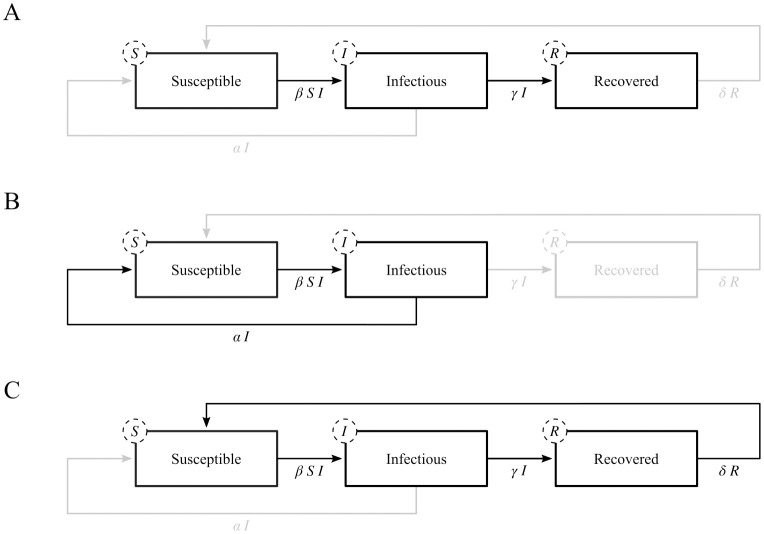
Summary of nested models. Flowcharts depicting three nested compartmental models that can be obtained by inducing sparsity among the parameters of the fully-connected SIRS model in Eq. (18). Panel (a) depicts the SIR model in Eq. (19). Panel (b) depicts the SIS model in Eq. (20). Panel (c) depicts the SIRS model in Eq. (21).

From a sparse learning perspective, treating the relevance of the three *rate* parameters *a*, γ, and δ as questionable ($N_{\alpha }=3$), gives a total of eight possible nested models ($2^{N_{\alpha }}$). Notably, among these eight models are three well-known nested models that can be obtained by setting the following combination of parameters to zero, as depicted by the flowcharts in Fig 2. The infection rate parameter β is not included among the questionable parameters as it is essential to the system dynamics and therefore considered to be an *a priori* relevant parameter. Further, as discussed below, it will later be treated as a *time-varying* parameter and thus be treated as an element of the augmented state vector.

a) A nested SIR model is obtained by eliminating the possibility of reinfection. In Fig 2A, this is achieved by setting *a* = 0, and δ=0.

$$\begin{array}{c} \frac{dS}{dt}=-\beta SI+aI+\delta R, \end{array}$$ (19a)

$$\begin{array}{c} \frac{dI}{dt}=\beta SI-aI-\gamma I, \end{array}$$ (19b)

$$\begin{array}{c} \frac{dR}{dt}=\gamma I-\delta R. \end{array}$$ (19c)

b) A nested SIS model is obtained by eliminating the recovered compartment. In Fig 2B, this is achieved by setting γ=0, and δ=0.

$$\begin{array}{c} \frac{dS}{dt}=-\beta SI+aI+\delta R, \end{array}$$ (20a)

$$\begin{array}{c} \frac{dI}{dt}=\beta SI-aI-\gamma I, \end{array}$$ (20b)

$$\begin{array}{c} \frac{dR}{dt}\;=\;\gamma I-\delta R. \end{array}$$ (20c)

c) A nested SIRS model is obtained by eliminating the possibility of reinfection without a period of temporary immunity. In Fig 2C, this is achieved by setting *a* = 0.

$$\begin{array}{c} \frac{dS}{dt}=-\beta SI+aI+\delta R, \end{array}$$ (21a)

$$\begin{array}{c} \frac{dI}{dt}=\beta SI-aI-\gamma I, \end{array}$$ (21b)

$$\begin{array}{c} \frac{dR}{dt}=\gamma I-\delta R. \end{array}$$ (21c)

Due to the inherent randomness in the transmission of diseases as well as the potential for temporal trends impacting the number of daily disease transmitting interactions, it is a reasonable modelling assumption to consider the infection rate as a time-varying parameter. Conversely, as *a*, γ, and δ represent average rates of recovery, they are less likely to vary significantly for large populations for the same strain of the disease. The time-varying parameter is modelled adopting the Wiener process model [16,41].


$$\begin{array}{c} \frac{d\beta }{dt}=q_{\xi }\xi (t), \end{array}$$ (22)

where ξ(t) is a Gaussian white noise process and $q_{\xi }$ represents the strength of the process noise. The time-varying parameter can be appended to the system states to construct the augmented state vector, and the process noise strength parameter can be included among the set of time-invariant parameters to be estimated.

Allowing the infection rate parameter β to vary in time while concurrently estimating the strength of the stochastic perturbation, $q_{\xi }$, affords each model the flexibility to fit the observations regardless of the assumed model form. This, however, makes it imperative to complement the model calibration by a model selection task such that the data-optimal model may be selected.

Upon augmenting the state vectors of the three nested models in the candidate set $ℳ=\{{ℳ}_{1},{ℳ}_{2},{ℳ}_{3}\}$ by appending the time-varying parameter to the original system states, the corresponding model operators, state vectors, and time-invariant parameters are given as:

a) ${ℳ}_{1}$: The nested SIR model has system states ${𝐮}_{k}=\{S_{k},I_{k},R_{k}\}$, time-varying parameter $\phi_{t(k)}=\{\beta_{k}\}$, and time-invariant parameters $\phi_{s}=\{\gamma ,q_{\xi },𝔼[\beta (t_{0})]\}$. For the augmented state vector ${𝐱}_{k}=\{S_{k},I_{k},R_{k},\beta_{k}\}$, the model operator is

$$\begin{array}{c} S_{k+1}=S_{k}+\Delta t\left(-\beta_{k}S_{k}I_{k}\right), \end{array}$$ (23a)

$$\begin{array}{c} I_{k+1}=I_{k}+\Delta t\left(\beta_{k}S_{k}I_{k}-\gamma I_{k}\right), \end{array}$$ (23b)

$$\begin{array}{c} R_{k+1}=R_{k}+\Delta t\left(\gamma I_{k}\right), \end{array}$$ (23c)

$$\begin{array}{c} \beta_{k+1}=\beta_{k}+\sqrt{\Delta t}\left(q_{\xi }\varepsilon_{k}\right). \end{array}$$ (23d)

b) ${ℳ}_{2}$: The nested SIS model has system states ${𝐮}_{k}=\{S_{k},I_{k}\}$, time-varying parameter $\phi_{t(k)}=\{\beta_{k}\}$, and time-invariant parameters $\phi_{s}=\{a,q_{\xi },𝔼[\beta (t_{0})]\}$. For the augmented state vector ${𝐱}_{k}=\{S_{k},I_{k},\beta_{k}\}$, the model operator is

$$\begin{array}{c} S_{k+1}=S_{k}+\Delta t\left(-\beta_{k}S_{k}I_{k}+aI_{k}\right), \end{array}$$ (24a)

$$\begin{array}{c} I_{k+1}=I_{k}+\Delta t\left(\beta_{k}S_{k}I_{k}-aI_{k}\right), \end{array}$$ (24b)

$$\begin{array}{c} \beta_{k+1}=\beta_{k}+\sqrt{\Delta t}\left(q_{\xi }\varepsilon_{k}\right). \end{array}$$ (24c)

c) ${ℳ}_{3}$: The nested SIRS model has system states ${𝐮}_{k}=\{S_{k},I_{k},R_{k}\}$, time-varying parameter $\phi_{t(k)}=\{\beta_{k}\}$, and time-invariant parameters $\phi_{s}=\{\gamma ,\delta ,q_{\xi },𝔼[\beta (t_{0})]\}$. For the augmented state vector ${𝐱}_{k}=\{S_{k},I_{k},R_{k},\beta_{k}\}$, the model operator is

$$\begin{array}{c} S_{k+1}=S_{k}+\Delta t\left(-\beta_{k}S_{k}I_{k}+\delta R_{k}\right), \end{array}$$ (25a)

$$\begin{array}{c} I_{k+1}=I_{k}+\Delta t\left(\beta_{k}S_{k}I_{k}-\gamma I_{k}\right), \end{array}$$ (25b)

$$\begin{array}{c} R_{k+1}=R_{k}+\Delta t\left(\gamma I_{k}-\delta R_{k}\right), \end{array}$$ (25c)

$$\begin{array}{c} \beta_{k+1}=\beta_{k}+\sqrt{\Delta t}\left(q_{\xi }\varepsilon_{k}\right). \end{array}$$ (25d)

where $\varepsilon_{k}$ is a Gaussian random variable with a mean of zero and unit standard deviation that arises due to the discretization of the Gaussian white noise process in Eq. (22). Note that $\varepsilon_{k}$ denotes the process noise, which should not be confused with the measurement noise, $\epsilon_{j}$, from Eq. (26).

### Measurement operator

Consider data generated from the realization of the SIRS model in Eq. (18) with parameters *a* = 0, γ=1/7, and δ=1/180 and initial conditions *S*(*t*_0_) = 0.999, *I*(*t*_0_) = 0.001 and *R*(*t*_0_) = 0, $\beta (t_{0})=0.18$. The chosen parameters correspond to an average infectious period of 7 days (1/γ), an average period of temporary immunity of 180 days (1/δ) wi*t*hou*t t*he possibility of immediate reinfection (*a* = 0). The initial states imply that 0.1% of the normalized population are initially infectious, the remainder are susceptible, and none have yet recovered. The initial value of the infection rate is best interpreted through the basic reproduction number, which gives $R_{0}=\beta (t_{0})/\gamma =1.26$.

The evolution of β(t) is driven by the realization of a Gaussian white noise process with strength $q_{\xi }=0.005$. The data are obtained by observing the infectious compartment *I*(*t*) once daily, corrupted by 5% multiplicative Gaussian noise [16]. This form of noise can be shown to reasonably approximate the widely used negative-binomial likelihood func*t*ion [18], ensuring that the noise strength is proportional to the number of infections, while conveniently working within the EKF framework adopted here. The general discrete measurement operator in Eq. (4) therefore becomes,


$$\begin{array}{c} {𝐝}_{j}=I_{d(j)}(1+\epsilon_{j}),\;\epsilon_{j}\sim 𝒩(0,0.01), \end{array}$$ (26)

where the index *d*(*j*) is to be interpreted as the *j*th observation of *I*_*k*_ occurring at time increment *k* = *d*(*j*). This choice of measurement operator represents daily reports of active cases, with measurement errors assumed to be proportional to the size of the infectious class. Considering newly reported cases as the measurements is also possible, whereby the observed quantity would be the rate of incidence, βSI, resulting in a measurement operator that is nonlinear with respect to the elements of the augmented state [42,43].

**Fig 3:**
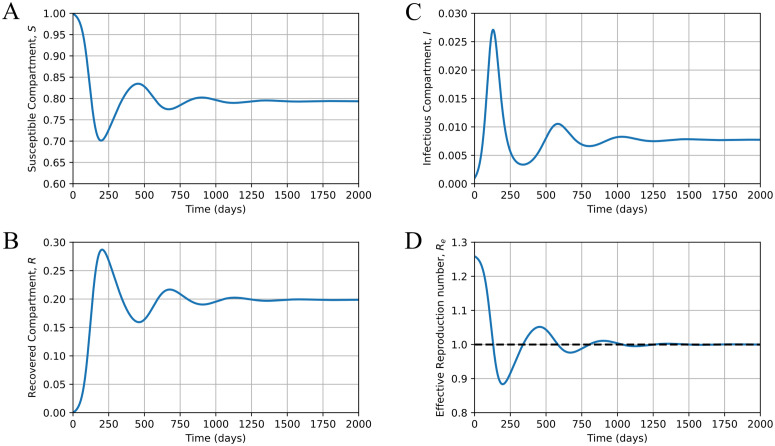
The long-term behaviour of an SIRS model. Panel (a) depicts the susceptible compartment, *S*(*t*), panel (b) depicts the infectious compartment, *I*(*t*), panel (c) depicts the recovered compartment *R*(*t*), and panel (d) depicts the effective reproduction number, *R*_*e*_(*t*).

The use of an SIRS model to generate the data imposes the possibility of reinfection, which is modelled by having individuals flow from the *R* compartment to the *S* compartment at a rate of δ, representing a period of temporary immunity equal to 1/δ days. For an SIRS model with time-invariant parameters, if immunity is temporary (δ>0) and the basic reproduction number *R*_0_ > 1, the SIRS model will result in an endemic equilibrium (recall, the basic reproduction number is computed as $R_{0}=\beta (t_{0})/\gamma$). This steady state solution in the limit of time t→∞ occurs as a balance is achieved between the three compartments, *S*(*t*), *I*(*t*), and *R*(*t*), where the effective reproduction number converges to $R_{e}(t)=1$, implying neither growth nor decay (recall, the effective reproduction number is computed as $R_{e}(t)=S(t)\beta (t)/\gamma$). Prior to the steady state solution, the transien*t* solu*t*ion consis*t*s of oscillatory behaviour in the infectious compartment, interpreted as multiple waves of infection, even in the case of a time-invariant infection rate parameter as depicted in Fig 3.

**Fig 4:**
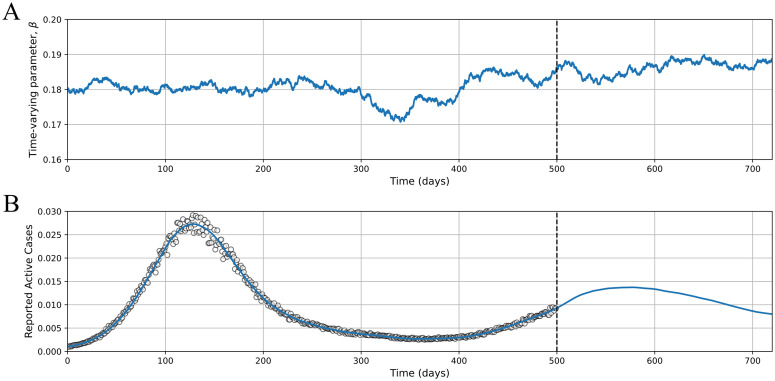
Synthetic data generated from an SIRS model. Panel (a) depicts the unobserved time-varying infection rate parameter, β, given by a realization of a Wiener process. Panel (b) depicts the resulting observed infectious compartment. The observations are corrupted by multiplicative Gaussian noise mimicking daily reports of active cases. Data points are indicated by white circles (∘) and the ground truth is given by a solid line (■).

Data are recorded up to the beginning of the second wave of infection at the 500 day mark, as depicted in Fig 4. This investigation assumes that the available data represent complete observations of the infectious compartment, presented in terms of their proportion of the total population. The remaining compartments are considered unobservable. The ground truth from which the synthetic data are generated is shown for an additional 220 days past the data cut-off, to depict the second peak of infection which is driven by waning immunity and time-varying trends in infection rate. Following parameter estimation, the three calibrated nested models are used to forecast future cases, which are compared against this ground truth in Fig 9.

**Fig 5:**
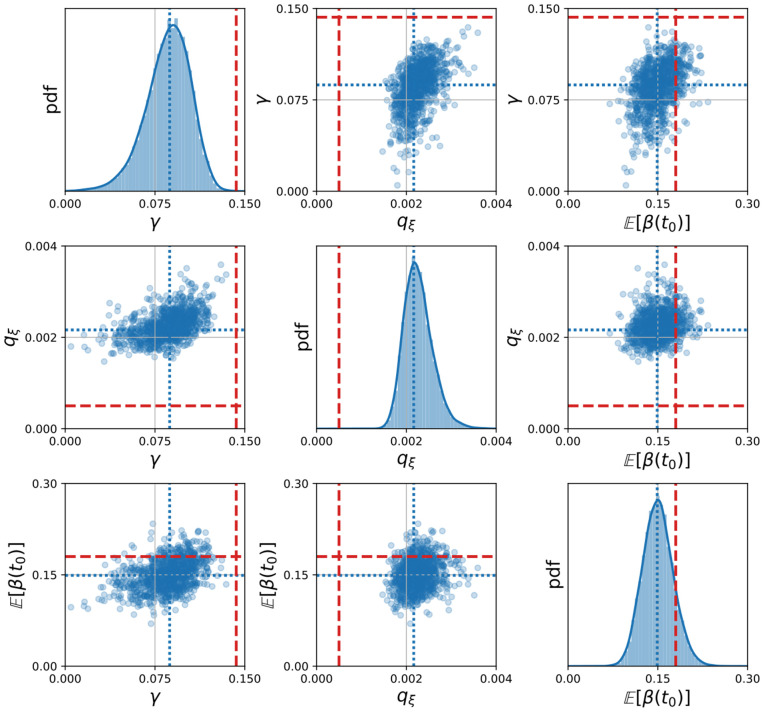
Marginal and joint parameter pdfs for the SIR model (${ℳ}_{1}$). Histogram and scatterplots of the pairwise joint MCMC samples of the time-invariant parameters, $\text{p}(\phi_{s}|𝒟,{ℳ}_{1})$. True parameter values are shown using dashed lines (■) and MAP estimates are shown using dotted lines (■).

**Fig 6:**
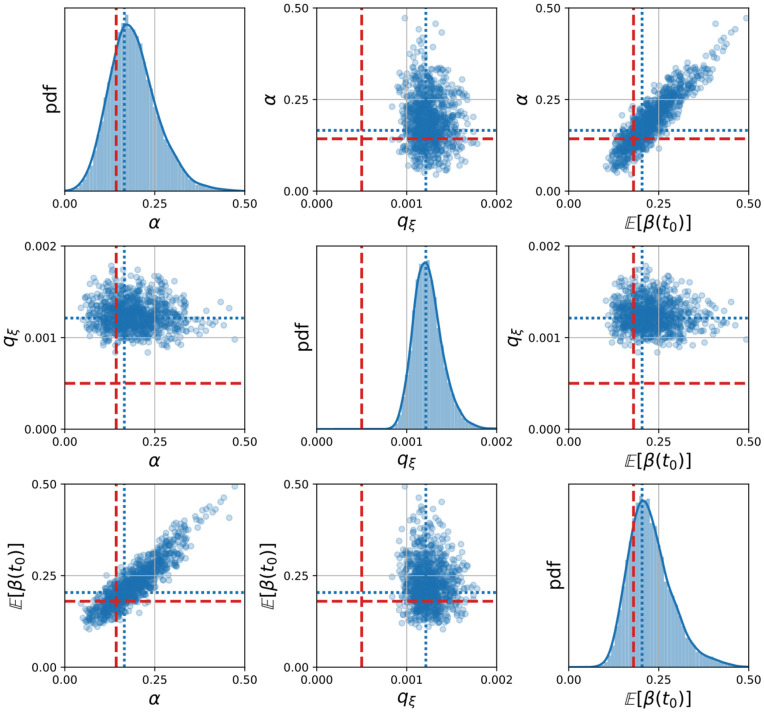
Marginal and joint parameter pdfs for the SIS model (${ℳ}_{2}$). Histogram and scatterplots of the pairwise joint MCMC samples of the time-invariant parameters, $\text{p}(\phi_{s}|𝒟,{ℳ}_{2})$. True parameter values are shown using dashed lines (■) and MAP estimates are shown using dotted lines (■).

**Fig 7:**
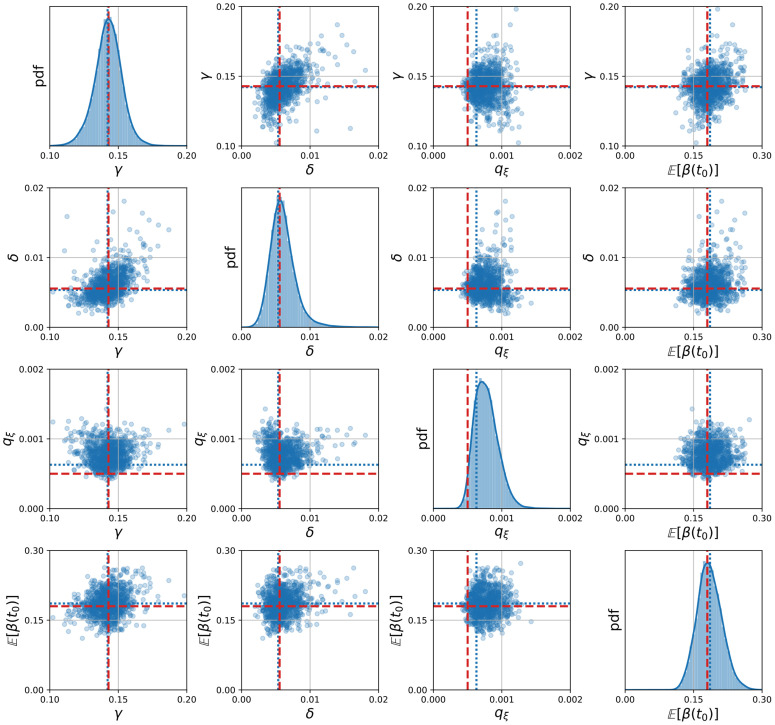
Marginal and joint parameter pdfs for the SIRS model (${ℳ}_{3}$). Histogram and scatterplots of the pairwise joint MCMC samples of the time-invariant parameters, $\text{p}(\phi_{s}|𝒟,{ℳ}_{3})$. True parameter values are shown using dashed lines (■) and MAP estimates are shown using dotted lines (■).

**Fig 8:**
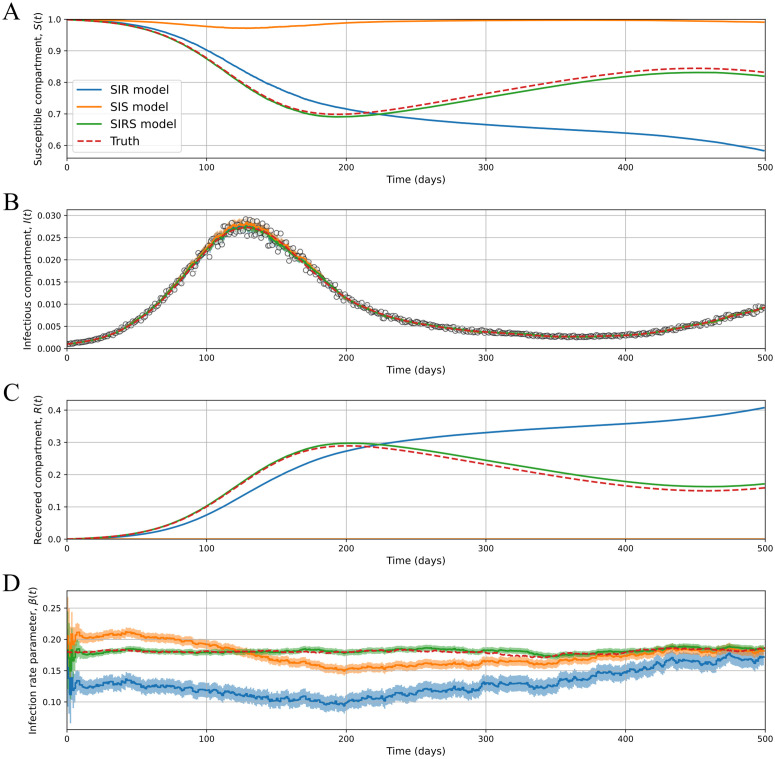
Summary of state estimation results. The conditional posterior augmented state pdfs $\text{p}({𝐱}_{1},\ldots ,{𝐱}_{K}|𝒟,\phi_{s}^{\text{map}},ℳ)$ are shown for the SIR, SIS, and SIRS models. Panel (a) depicts the susceptible compartment, *S*, panel (b) depicts the infectious compartment *I*, alongside the noisy measurements, panel (c) depicts the recovered compartment, *R*, and panel (d) depicts the time-varying infection rate parameter, β. The ground truth is shown using a dashed line (■), the mean estimates are indicated by a solid lines (■ for the SIR model, ■ for the SIS model, and ■ for the SIRS model), and the correspondingly coloured shaded areas reflect the mean ± 1.96 standard deviations.

### Effect of model uncertainty and misspecified models

The three nested models of interest, $ℳ=\{{ℳ}_{k}{\}}_{k=1}^{3}$, in Eqs. (23)–(25) are calibrated using the same data, $𝒟=\{{\text{d}}_{j}{\}}_{j=1}^{500}$, given by the measurement operator in Eq. (26), providing direct observations of the infectious compartment.

The time-invariant parameter estimates for the SIR model are summarized in Fig 5, those for the SIS model are summarized in Fig 6, and those for the SIRS model are summarized in Fig 7. In all three cases, the histograms of the MCMC samples and the marginal pdfs constructed using KDE depict well-sampled unimodal posterior pdfs, indicating well-behaved inference. The posterior pdfs of the time-invariant parameters of the SIR model (Fig 5) and of the SIS model (Fig 6) do not capture the true parameter values. This is not a shortcoming of the estimation procedure, but rather a consequence of erroneous dynamics described between the assumed model structure and the data-generating process. The converse is observed when the SIRS model is used. As the data-generating model, the true value of the time-invariant parameters are all captured within the support of the posterior pdfs shown in Fig 7.

Due to the joint statistical nature of their estimation, the pdfs of the time-invariant parameters in Figs 5–7 should be interpreted alongside the augmented state estimates in Fig 8. For simplicity, these marginal state pdfs are conditioned on the MAP estimate of the conditional posterior of the time-invariant parameters and superimpose the depiction of the three respective compartmental models, the SIR model (■), the SIS model (■), and the SIRS model (■). Note from Fig 8B, each of the three models, augmented by a time-varying infection rate parameter, are capable of reproducing the dynamics of the observed compartment. However, as highlighted in the remaining panels which depict the susceptible compartment (Fig 8A), the recovered compartment (Fig 8C) and the time-varying infection rate parameter (Fig 8D), the overall system dynamics are quite disparate. Only the SIRS model, having the same form as the data-generating model, follows the ground truth in all four panels (dashed line ■).

The flexibility that is afforded to the model by permitting the infection rate parameter to vary in time allows for the accurate inference of the infectious compartment in Fig 8 for each of the three models. The measurement operator in Eq. (26), which defines the reported active case data as direct measurements of the infectious compartment, leads to the precise estimates of that element of the state vector in Fig 8B. The joint estimation of the augmented state vector and the time-invariant parameters provides a robust mechanism for parameter inference, however, the inferred states and parameters are of course conditioned on the chosen model. The use of synthetic data in the current numerical experiments enables the comparison of the inferred states and parameters for each model against their known ground truth values.The SIRS model, which shares the structure of the data-generating process, yields augmented state estimates that exhibit good visual agreement with the ground truth across all panels in Fig 8. In the absence of a known ground truth, a systematic model selection procedure would be necessary to quantitatively evaluate the competing models rather than relying on qualitative visual agreement. This motivates the sparse learning exercise that follows.

**Fig 9:**
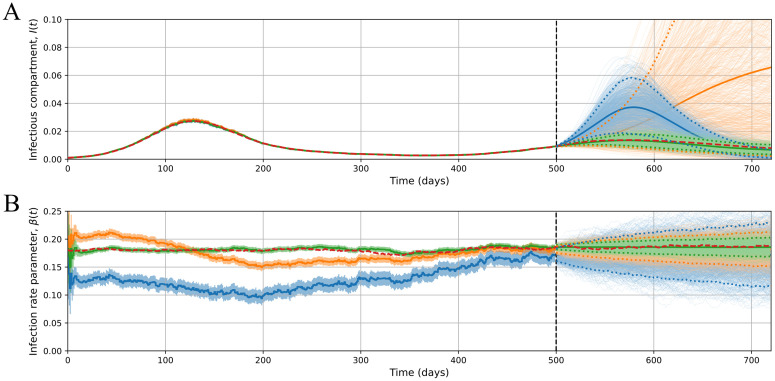
Summary of model forecasts. Marginal state pdfs for synthetic data generated from an SIRS model, conditioned on the MAP estimate of the conditional posterior of the time-invariant parameters. Panel (a) depicts the infectious compartment *I*, and panel (b) depicts the time-varying infection rate parameter, β. The ground truth is shown using a dashed line (■), the mean estimates are indicated by a solid lines (■ for the SIR model, ■ for the SIS model, and ■ for the SIRS model). The dotted lines depict the bounds of inner 90th percentile of the non-Gaussian forecasted state pdf.

Fig 9 depicts the forecasts of two critical quantities of interest: i) the infectious compartment in Fig 9A, and ii) the infection rate parameter in Fig 9B. While the infectious curve was well captured while data was being assimilated, after the data-cutoff at day 500, the SIR and SIS models both fail to capture the ground truth as time progresses. Conversely, the SIRS model succeeds in describing the long-term dynamics of the system. Beyond the data cut-off at day 500, 1000 Monte Carlo (MC) sample realizations of the infection rate are propagated through the respective nonlinear models to provide an ensemble-based approximation of the forecast mean and uncertainty. The dotted lines depict the estimates of the 5th and 95th percentiles, illustrating the bounds of inner 90th percentile of the non-Gaussian forecasted state pdf for each model. Note that all three model forecasts begin from similar initial conditions at day 500. After the data cut-off, the ensemble predictions of the three models in Fig 9A begin to deviate both in terms of their central tendency and variance. The principal drivers for the observed difference between the three ensembles are i) the distinct dynamics prescribed by each of the three respective models, and ii) the influence of the realizations of the Wiener process model of the infection rate parameter in Fig 9B as they are propagated through the nonlinear models. Because the structure of the SIRS model (■) describes the same dynamics as the data-generating model, it required very little artificial process noise to track the latent infection rate parameter, resulting in forecasts with lower variance. The associated ensemble estimate for the infectious compartment therefore tracks the ground truth accurately and with relatively low uncertainty. The SIS model (■) captures the dynamics of reinfection of the data-generating model, but fails to reflect temporary immunity. As a result of the mischaracterization of the dynamics, more significant variations in the infection rate parameter are required such that the dynamics of the infectious compartment matches the observations. Achieving the larger time-variation necessitates a higher level of artificial noise. The resulting increase in the variance of the forecast of the infection rate parameter, produces both faster exponential growth and decay at the extremes when propagated through the nonlinear SIS model. As a result, these predictions envelope the SIRS model ensemble predictions and capture the ground truth with higher mean error and much larger uncertainty. The SIR model (■) lacks a mechanism for reinfection and therefore requires the highest process noise strength among the three models to reproduce the observed infection data. Unlike the SIS model, the SIR model forecasts do not envelope the SIRS model predictions of the infectious compartment and, for much of the forecast horizon, it does not capture the ground truth. The reinfection mechanism which replenishes the susceptible compartments of the SIS models and SIRS models naturally produce multiple transient waves of infection, as the effective reproduction number oscillates above 1 (recall $R_{e}(t)=S(t)\beta (t)/\gamma$). In the SIR model, the susceptible compartment *S*(*t*) is monotonically decreasing, and γ is a time-invariant parame*t*er. Hence, to produce secondary waves of infection, the infection rate (relative to γ) must increase artificially to offset the depletion of the susceptible compartment. The distinct peak in the SIR model can be attributed to the depletion of the susceptible compartment which, through the dynamics imposed by the model structure, causes the uncertainty in the ensemble estimate of the infections to decrease, despite the continual growth of the uncertainty in the infection rate parameter.

The ability of all candidate models to accurately reproduce the observed dynamics before the data cut-off in Fig 9, followed by a deviation in the forecasted trajectories afterwards, further underscores the need for model selection. Clearly, from Fig 8, if one were to observe one of the other elements of the state vector, the optimal model would be self-evident. This partially explains why model discovery approaches that assume fully observed states such as [1,7] are capable of considering higher-dimensional, unconstrained problems, with practical limitations arising primarily from data availability. In some applications, additional measurement devices may be utilized to obtain a complete observations of the system states. However, in applications such as the current example of infectious disease modelling, testing measures typically only provide information on a subset of disease states. It is not feasible to assess the compartment membership of an entire population simultaneously, nor is it possible to determine if non-infectious individuals possess immunity, and if so, the duration.

As shown in Fig 9, the presence of stochasticity in the system dynamics enables all three of the competing compartmental models $\{{ℳ}_{i}{\}}_{i=1}^{3}$ to achieve comparable data fit under a partially observed system. In contrast to sparse regression approaches that may assume full state observability and deterministic dynamics, the proposed solution for model structure inference formulates model structure inference as a sparse learning problem that identifies the data-optimal model through Type-II MAP estimation. Within the nested model formulation, the induced sparsity in the parameter space directly dictates the coupling structure of the of the optimal compartmental model, thereby encoding the interconnectivity of the modelled compartments. Note, however, that data-optimality does not guarantee correctness. As an evidence-based model selection approach, it seeks the optimal trade-off in average data fit and model complexity, which may not correspond to the true data-generating process.

### Model structure inference

As alluded to during the problem description, the task of discovering the optimal nested compartmental model to describe the observed dynamics can be approached using sparse learning on an over-parameterized compartmental model, whereby different combinations of sparsity structures among the parameters represent different unique compartmental models.

The over-parameterized compartmental model obtained by coupling Eqs. (18) and (22) has system states ${𝐮}_{k}=\{S_{k},I_{k},R_{k}\}$, time-varying parameter $\phi_{t(k)}=\{\beta_{k}\}$, and time-invariant parameters $\phi_{s}=\{a,\gamma ,\delta ,q_{\xi },𝔼[\beta (t_{0})]\}$. For the augmented state vector ${𝐱}_{k}=\{S_{k},I_{k},R_{k},\beta_{k}\}$, the model operator is


$$\begin{array}{c} S_{k+1}=S_{k}+\Delta t\left(-\beta_{k}S_{k}I_{k}+aI_{k}+\delta R_{k}\right), \end{array}$$ (27a)

$$\begin{array}{c} I_{k+1}=I_{k}+\Delta t\left(\beta_{k}S_{k}I_{k}-aI_{k}-\gamma I_{k}\right), \end{array}$$ (27b)

$$\begin{array}{c} R_{k+1}=R_{k}+\Delta t\left(\gamma I_{k}-\delta R_{k}\right), \end{array}$$ (27c)

$$\begin{array}{c} \beta_{k+1}=\beta_{k}+\sqrt{\Delta t}\left(q_{\xi }\varepsilon_{k}\right). \end{array}$$ (27d)

The associated NSBL setup is summarized in Table 1. The problem setup considers augmented state ${𝐱}_{k}=\{S_{k},I_{k},R_{k},\beta_{k}\}$, comprised of the original system states, (i.e., the compartments, ${𝐮}_{k}=\{S_{k},I_{k},R_{k}\}$) and time-varying parameter $\phi_{t(k)}=\{\beta_{k}\}$. While it is not known whether the infection rate varies in time *a priori*, modelling it as such while concurrently estimating the process noise strength $q_{\xi }$ permits the flexibility to infer the underlying nature of the parameter β.

**Table 1:** Sparse learning setup. Three-dimensional sparse learning setup for synthetic infection data from an SIRS model. Time-varying parameter $\left\{\beta_{k}\right\}$ is appended to the original state vector, *a priori* relevant parameters $\left\{q_{\xi },𝔼[\beta (t_{0})]\right\}$ are assigned known priors and questionable parameters {a,γ,δ} are assigned ARD priors.

System state vector	u_*k*_ = $\begin{array}{c} \left\{S_{k},I_{k},R_{k}\right\} \end{array}$
Augmented state vector	x_*k*_ = $\left\{S_{k},I_{k},R_{k},\beta_{k}\right\}$
Time-varying parameter(s)	$\phi_{t(k)}$ = $\left\{\beta_{k}\right\}$
*A priori* relevant parameters	$\phi_{\text{-}\alpha }$ = $\left\{q_{\xi },𝔼[\beta (t_{0})]\right\}$
Known prior, $\text{p}(\phi_{\text{-}\alpha })$	$\text{Lognormal}(q_{\xi }|0.001,1.0)\text{ Lognormal}(𝔼[\beta (t_{0})]|0.2,0.25)$
Questionable parameters	$\phi_{\alpha }$ = {a,γ,δ}
ARD prior, $\text{p}(\phi_{\alpha }|\alpha )$	$𝒩(a|0,\alpha_{1}^{-1})𝒩(\gamma |0,\alpha_{2}^{-1})𝒩(\delta |0,\alpha_{3}^{-1})$

**Fig 10:**
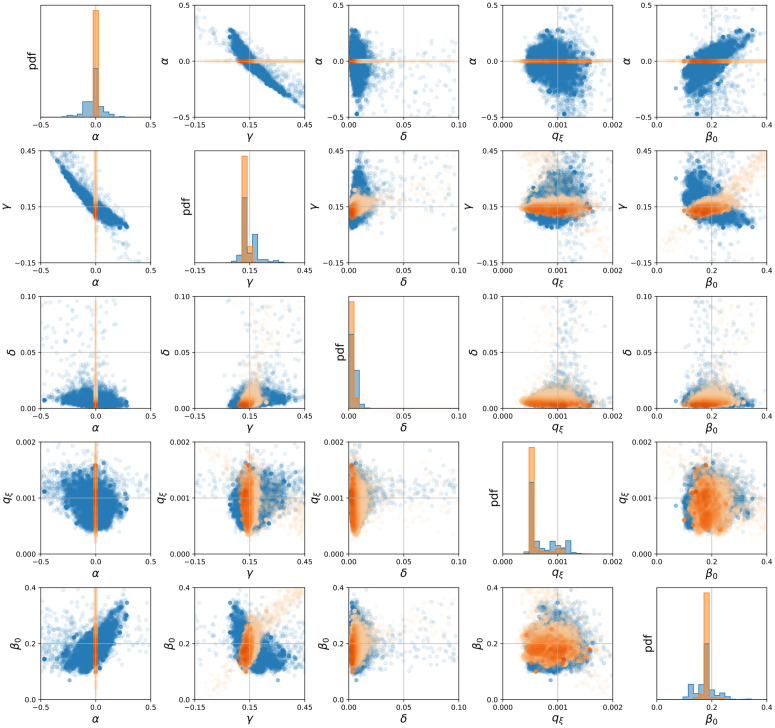
Summary of sparse learning results. Histogram and scatterplots of the pairwise joint MCMC samples of the time-invariant parameters, before sparse learning, $\mu^{\left(k\right)}$ (■), and after sparse learning, ${\text{m}}^{\left(k\right)}$ (■). The intensity of the colour indicates the relative kernel weights.

The estimation results for the time-invariant parameters in the fully-connected SIRS model are shown in blue in Fig 10. The marginal parameter posterior pdfs are depicted as histograms of the MCMC samples, while pairwise joint pdfs are depicted by scatterplots. The results before sparse learning are shown in blue (■), with each sample carrying equal weight. Note that the joint statistical properties between parameters *a* and γ, being the two parameters controlling the flow out of the observed compartment, *I*, results in a significantly more arduous task of generating MCMC samples from the parameter posterior. The effect of the optimal hyperparameter selection through sparse learning can be visualized by the shifting of the coordinates of the samples and the adjustment of their weights, shown in orange (■). After sparse learning, the histograms are constructed considering the shifted samples and their weights. Similarly, in the joint plots, the scatterplot of the shifted samples is superimposed on the original MCMC samples, with their relative weights reflected in the colour intensity of the point.

**Fig 11:**
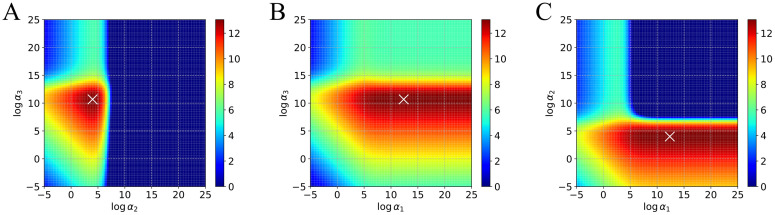
Log evidence as a function of the hyperparameters. Two-dimensional section view of the log evidence depicting the optimal hyperparameters (×), panel (a) depicts the evidence for $\log\alpha_{1}=12.36$, panel (b) depicts the evidence for $\log\alpha_{2}=4.00$, and panel (c) depicts the evidence for $\log\alpha_{3}=10.67$.

The Gamma hyperpriors, defined in in Eq. (14), are each assigned shape and rate $r_{i}=s_{i}=e^{-20}$. These values were chosen to be sufficiently small to minimize the influence of the hyperprior on the location of the optimum of the NSBL objective function in Eq. (16), thereby rendering the solution effectively equivalent to evidence maximization. The optimal values of the hyperparameters were found to be $\log\alpha_{1}=12.39$ (corresponding to α), $\log\alpha_{2}=4.00$ (corresponding to γ), and $\log\alpha_{3}=10.67$ (corresponding to δ). The log evidence is plotted in a pairwise fashion in Fig 11 along with the coordinates of the global optimum. These plots provide pairwise sectional views of the log evidence surface as a function of two hyperparameters with the remaining hyperparameter fixed to its optimal value.

A scale-independent metric, referred to as the *relevance indicator* [8–10], lends a practical interpretation to the optimal values of the hyperparameters. For each parameter, this metric considers the ratio of its posterior precision to its prior precision, providing a value between 0 and 1 indicating irrelevance or relevance of that parameter, respectively. For the exact expression, see Eq. (23) in Ref. [9]. The associated relevance indicator values for parameter *a* is 0.0034, for γ is 0.9982, and for δ is 0.9781. These values imply the relevance of parameters γ and δ and the irrelevance of parameter *a*. This illustrates the purpose of the relevance indicator. Whereas the parameters *a* and δ have similar optimal values of $\log\alpha_{i}$, the difference in scale of the parameters results in differing conclusions regarding their relevance. This combination of relevant and irrelevant parameters ultimately correctly identifies the data-generating SIRS model, characterized by parameter *a* being equal to zero, with non-zero values for γ and δ. The inferred sparsity structure given by the relevance indicators correspond to that of the nested SIRS model, as depicted in Fig 2C.

Table 2 summarizes the mean and standard deviation of the parameters of the fully-connected SIRS model before and after sparse learning (see Fig 10). While the mean of the pdf may not be an ideal indicator of central tendency, due to the non-Gaussian features of the parameter posterior pdf, here it is used to show the convergence of the sparse learning results towards the inference results obtained using the data-generating model. Furthermore, the reporting of the standard deviation is intended to demonstrate the reduction in uncertainty that results in the automatic pruning of redundant parameters as well as the associated reduction in uncertainty among relevant parameters as a consequence of removing irrelevant parameters. This reduction in uncertainty is the combined effect of the updated posterior kernel weights, means and covariance conditioned on the optimal hyperparameters. The mean of the redundant parameter, *a*, is forced towards zero by the restrictive prior, and similarly the uncertainty surrounding that mean estimate is reduced by a factor of 44.73. Additionally, the optimal prior selection reduces parametric uncertainty among the relevant parameters, with γ seeing a reduction by a factor of 2.958 and δ by 35.95. This follows directly from the conditional representation of the GMM of the parameter posterior as a function of the hyperparameters in Eq. (17). Once the hyperparameters are optimized, the data-optimal sparsity structure is enforced automatically, and the parameter estimates of the inferred nested model are reflected in the joint statistical properties encoded in the GMM. The joint statistical properties between the questionable parameters, $\phi_{\alpha }=\{a,\gamma ,\delta \}$ can be visualized by the top left 3×3 block of scatterplots in Fig. (10).

**Table 2:** Summary of sparse learning results. The mean and standard deviations of the time-invariant parameters are reported for before and after sparse learning. These results are compared to the original results obtained from the SIRS model in Fig 7.

Parameter	*a*	γ	δ	$q_{\xi }$	$𝔼[\beta (t_{0})]$
Truth	0	1.429$\times 10^{-1}$	5.556$\times 10^{-3}$	5.000$\times 10^{-4}$	1.800$\times 10^{-1}$
Optimal hyperparameter $\log{\alpha_{i}}^{\text{map}}$	12.39	4.00	10.67	–	–
Relevance indicator	0.0034	0.9982	0.9781	–	–
Mean (pre-NSBL)	−1.297$\times 10^{-2}$	1.496$\times 10^{-1}$	7.860$\times 10^{-3}$	7.257$\times 10^{-4}$	1.780$\times 10^{-1}$
Mean (post-NSBL)	5.652$\times 10^{-5}$	1.353$\times 10^{-1}$	4.671$\times 10^{-3}$	6.317$\times 10^{-4}$	1.776$\times 10^{-1}$
Mean (SIRS)	–	1.426$\times 10^{-1}$	6.182$\times 10^{-3}$	7.737$\times 10^{-4}$	1.846$\times 10^{-1}$
Standard deviation (pre-NSBL)	9.224$\times 10^{-2}$	6.066$\times 10^{-2}$	3.773$\times 10^{-2}$	2.595$\times 10^{-4}$	4.012$\times 10^{-2}$
Standard deviation (post-NSBL)	2.062$\times 10^{-3}$	2.050$\times 10^{-2}$	1.050$\times 10^{-3}$	1.844$\times 10^{-4}$	1.498$\times 10^{-2}$
Standard deviation (SIRS)	–	10.10$\times 10^{-3}$	4.018$\times 10^{-3}$	0.1639$\times 10^{-3}$	26.79$\times 10^{-3}$

## Conclusion

### Model structure inference

Embedding a sparse learning algorithm within a Bayesian computational framework for stochastic dynamical systems allows the concurrent estimation of system states, time-varying parameters, and time-invariant parameters while automatically inferring the data-optimal nested model structure. Modelling parameters as time-varying and jointly estimating the associated process noise strength provides robust state and parameter inference under minimal assumptions about the underlying nature of the time-varying parameters. This flexibility is advantageous in well-specified models, however, it also permits misspecified models to reproduce observed dynamics while latent states and parameters exhibit significant error. Traditional model selection approaches can mitigate risks associated with model uncertainty, with NSBL playing the role of data-optimal model selection, operating on regularized evidence optimization.

In the numerical example, embedding NSBL within the broader Bayesian computational framework correctly identified the data-generating model, evidenced by the relevance indicators. Inferring the optimal sparse representation of model parameters in a coupled system of differential equations inherently reveals the optimal coupling structure. This model structure inference is computationally efficient, achieved through the use of sparsity-inducing Gaussian ARD priors in a semi-analytical hierarchical Bayesian framework. Highly non-Gaussian entities are represented as mixture of multivariate Gaussians, enabling tractable optimization of the regularized model evidence through semi-analytical functions of the ARD prior and GMM kernel parameters. This process reveals the data-optimal sparse representation of the time-invariant model parameters, implementing a numerical Occam’s razor, balancing model complexity and average data-fit.

Model structure inference via the NSBL algorithm achieved goals similar to traditional model discovery approaches, while relaxing limiting constraints of regularized regression methods. As NSBL is formulated for arbitrary mappings from inputs to outputs, it can be applied directly within established sparse model discovery frameworks with minimal modifications. However, its success in such settings critically depends on the ability to compute an informative likelihood function in addition to any information encoded in specified prior pdfs. Whereas model discovery in dynamical systems typically relies on full state observations, thereby providing rich information for identifying the governing equations, NSBL relies only on the availability of the likelihood function, which can be constructed from partial state observations. This enables the broader application of NSBL to practical problems involving incomplete and noisy data, but may limit the extent to which the sparsity can be reliably identified due to reduced limited information relative to fully observed systems. Nevertheless, the reliance on the likelihood function enables its native extension to stochastic dynamical systems with quantified uncertainty. To combat this, unlike many model discovery studies, which consider large libraries of candidate nonlinear terms, this work focused on a reduced, physically constrained set of nested candidate models. By embedding prior expert knowledge into the problem setup, the framework avoided starting from an intentionally naïve representation, instead leveraging inference to successfully identify the optimal model structure within a principled and informed search space.

### Infectious disease modelling

Through a low-dimensional, practical example in infectious disease modelling, it was shown that the optimal nested model structure could be successfully rediscovered from partial observations of the system states. The data-optimal model structure was inferred concurrently with the augmented state and model parameters. Synthetic data were intentionally used to enable validation of the estimated states, parameters, and sparsity structure against a known ground truth. Future work should focus on i) validating this approach in retrospective analyses with public health data, benchmarked against consensus clinical quantities, ii) exploring its scalability to more complex compartmental models, and iii) extending the proposed model structure inference framework to applications outside of infectious disease modelling and public health.

While this paper foremost presents a general framework for stochastic dynamical systems, the numerical example demonstrates the potential for clinical and public health translation. From a clinical and health-systems perspective, the ability to capture time-varying parameters enables timely identification of shifts in disease transmission or severity patterns, which may reflect emerging variants, behavioural changes, or modifications in clinical management. Principally, within a compartmental model framework, forecasts fundamentally rely on the number of active infections, the infection rate, and the number of susceptible individuals. While active infections are a regularly observed quantity in the numerical example, the infection rate and susceptible population remain latent elements of the augmented state. The nonlinear interaction of these three states govern future disease dynamics. Similarly, critical indicators such as the effective reproduction number are nonlinear combinations of latent states and model parameters. As demonstrated, misspecified models may accurately reproduce the observed dynamics, in this case active infections, while exhibiting substantial errors in unobserved states. Such errors propagate into misleading forecasts and potentially increased uncertainty. Improving the accuracy and uncertain quantification of predicted case counts could therefore support better-informed public health measures and enable more efficient healthcare resource allocation during rapidly evolving outbreaks. The results further show that, under misspecified models, parameter estimates associated with key clinical quantities, such as the average duration of infection or immunity, may deviate significantly from their true values. This highlights the risks of drawing clinical or policy conclusions from inadequately specified population-level models and underscores the importance of model selection in epidemiological forecasting.
